# An Adult-Onset Case of Porokeratotic Eccrine Ostial and Dermal Duct Nevus (PEODDN) in a Previously Healthy 28-Year-Old Indian Male: A Case Report

**DOI:** 10.7759/cureus.88722

**Published:** 2025-07-25

**Authors:** Shehab Aldhafiri, Ali Alroumi, Sameh Al Mushara, Shaima Ahmad

**Affiliations:** 1 Dermatology, Al Jahra Hospital, Al Jahra, KWT; 2 Dermatopathology, As'ad Al-Hamad Dermatology Center, Kuwait City, KWT

**Keywords:** acitretin, dermatology, parakeratosis, peoddn, porokeratotic nevus, skin biopsy, sweat gland hamartoma

## Abstract

A 28-year-old Indian male with no previous medical or dermatological history presented with mildly pruritic linear skin lesions over the right anterior chest and arm. The lesions had been gradually progressing over the course of six years. Examination revealed multiple confluent, reddish-brown papules following Blaschko’s lines, along with punctate hyperkeratotic papules over the right hand, with sparing of the mucosa, scalp, and nails. Histopathological analysis demonstrated a cup-shaped epidermal invagination filled with a parakeratotic column, absence of the granular layer, and dyskeratotic keratinocytes, with an eccrine duct located at the base of the lesion - indicating an acrosyringial origin. These findings confirmed the diagnosis of porokeratotic eccrine ostial and dermal duct nevus (PEODDN). PEODDN is a rare hamartomatous disorder with eccrine differentiation. While it usually presents at birth or during childhood, adult-onset cases have been documented - particularly along Blaschko’s lines. The condition may present with porokeratotic plaques or comedo-like plugs on acral sites, and our case uniquely exhibited both features. Although its pathogenesis remains unclear, several theories suggest abnormal keratinization, structural defects in eccrine ducts, and possible genetic mutations. PEODDN may occasionally be associated with systemic or neoplastic conditions. Treatment options remain variable, and while no standard therapy offers complete resolution, our findings support the potential role of oral retinoids in improving lesion appearance and symptoms in adult-onset cases. Further evaluation of systemic retinoid therapy in PEODDN is warranted.

## Introduction

Porokeratotic eccrine ostial and dermal duct nevus (PEODDN) is a rare hamartomatous condition involving the eccrine sweat ducts, characterized histologically by corned lamellae overlying eccrine acrosyringia. It is now considered part of the broader spectrum of porokeratotic adnexal ostial nevus (PAON), which includes porokeratotic eccrine and hair follicle nevus (PEHFN). This condition clinically manifests as multiple keratotic papules or plaques arranged in either a linear or Blaschkoid pattern, typically presenting in early life and most often affecting the palms and soles. The condition may be asymptomatic or present with mild pruritus or pain. Moreover, PEODDN may be associated with other conditions, including dermatological diseases like squamous cell carcinoma or systemic diseases like hyperthyroidism, sensory polyneuropathy, or developmental delays [1]. It is important to note that, while the condition is rare, adult-onset presentations are distinctly uncommon - particularly when distributed along Blaschko’s lines [2].

This case describes a 28-year-old, previously healthy Indian male who presented six years ago with mildly pruritic, reddish-brown papules in a linear distribution over the right anterior chest and upper extremity, without associated systemic symptoms, relevant personal or family history of dermatologic disease, or abnormalities in lab results - including complete blood count, metabolic panel, and thyroid function tests. This report further aims to provide a detailed account of the patient’s clinical presentation, laboratory investigations, and histopathological findings.

## Case presentation

A previously healthy 28-year-old Indian male presented to the clinic with mildly pruritic skin lesions over the right anterolateral aspects of the chest and arm. These lesions began and progressed over the past six years, with no systemic involvement or personal or familial history of other skin diseases.

**Figure 1:**
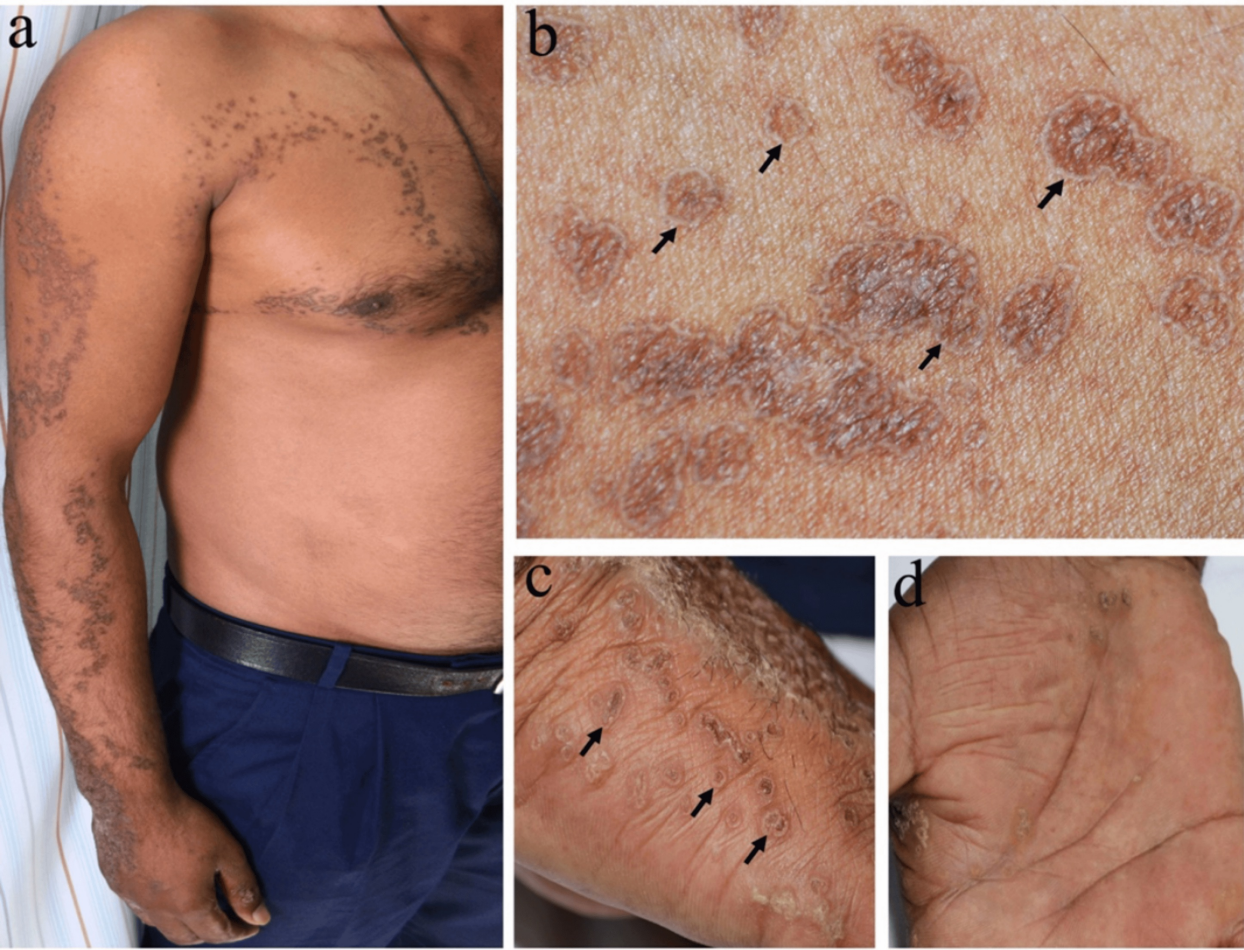
Clinical examination Multiple, closely related, and confluent reddish-brown porokeratotic papules involving the right anterior chest and upper extremity along Blaschko’s lines (a). Characteristic hyperkeratotic borders are seen in the close-up views, as highlighted by the arrows (b and c). Multiple palmar pits admixed with warty lesions (d).

On clinical examination, he had multiple closely related and confluent reddish-brown porokeratotic papules in a linear configuration involving the right anterior chest and upper extremity, following Blaschko’s lines. There were multiple punctate hyperkeratotic papules on the dorsal, palmar, and lateral aspects of the right hand as well (Figure 1).

However, the mucous membranes, teeth, and scalp were free of any lesions. As such, the differential diagnosis included linear porokeratosis, linear verrucous epidermal nevus, linear lichen planus, lichen striatus, and linear psoriasis.

**Figure 2:**
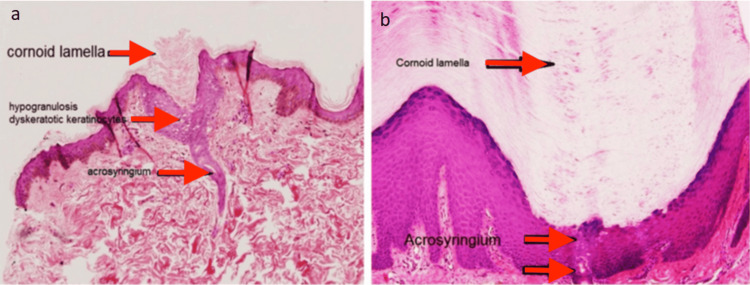
Histopathology images Skin biopsies from the right anterior chest (a) and right palm (b) show parakeratotic columns (cornoid lamellae) in association with eccrine ostium and duct dilation, along with hyperplasia of the acrosyringium and deeper eccrine duct.

Biopsies were taken from the right anterior chest and right thumb. Histopathology showed a cup-shaped invagination in the epidermis filled with a parakeratotic column and associated with the absence of the granular layer and the presence of dyskeratotic keratinocytes underneath the column. An eccrine duct was found at the base of the invagination, with an acrosyringial origin (Figure 2).

Based on both the clinical and histological findings, we diagnosed PEODDN. The presence of cornoid lamellae directly over eccrine acrosyringia was characteristic, and the absence of other features - such as lichenoid inflammation seen in lichen planus and lichen striatus; papillomatosis or prominent epidermal hyperplasia seen in verrucous epidermal nevus; or psoriasiform epidermal hyperplasia with Munro microabscesses seen in psoriasis - helped exclude other conditions.

The patient was started on topical corticosteroids with salicylic acid for one month, but no improvement was seen. The patient was later started on 25 mg of acitretin daily, which resulted in significant improvement, as evidenced by flattening of the lesions and decreased scaling.

## Discussion

Adult-onset PEODDN is a rare hamartoma with eccrine differentiation. Most reported cases show palm or sole involvement, but a few have described lesions on other areas of the body along Blaschko’s lines. Onset may occur from birth and early childhood to late adolescence, with no sex predilection. PEODDN typically presents in one of two patterns: porokeratotic lesions that coalesce into plaques along Blaschko’s lines on the trunk or extremities, or linear punctate pits with comedo-like plugs on the palms or soles. Our patient exhibited both features [1,2].

The condition was first described by Marsden et al. in 1979 [3], with further characterization by Abell and Reed in 1980 [4]. Although the exact pathogenesis remains unclear, several hypotheses have been proposed. Initially, it was believed to represent an epidermal invagination composed of a dilated, keratin-plugged acrosyringial duct with a dermal continuation from the base [5]. Stoof et al. proposed that the lack of carcinoembryonic antigen (CEA) expression by acrosyringial cells reflected abnormal structural development [6]. Masferrer et al. suggested it originates from a localized keratinization abnormality, supported by CEA positivity along the ductal lumina through the parakeratotic column of the cornoid lamella [7]. Wang et al. later hypothesized that increased proliferation of the basal layer beneath the cornoid lamella contributes to abnormal keratinizing epidermal invagination, confirmed by Ki-67 staining [8]. More recently, some researchers have proposed that PEODDN represents a mosaic form of keratitis-ichthyosis-deafness (KID) syndrome, caused by a mutation in GJB2 (gap junction protein beta 2), which encodes the gap junction protein Cx26 (connexin-26) [9].

PEODDN has rarely been associated with other dermatologic conditions such as alopecia, onychodysplasia, Bowen’s disease, and squamous cell carcinoma. There are also reports of linear psoriasis coexisting with PEODDN, possibly due to somatic mutations within a gene implicated in the polygenic predisposition to psoriasis [10]. Additionally, associations with neurological and endocrine disorders - including deafness, hemiparesis, sensory polyneuropathy, seizures, and hyperthyroidism - have been noted [2].

Although several treatment options have been tried - such as surgical excision, phototherapy, cryotherapy, electrocautery, CO₂ laser, and topical agents including keratolytics, retinoids, corticosteroids, calcipotriol, and anthralin - none have proven definitively curative [2]. Our patient was treated with 25 mg of acitretin daily for four months, resulting in significant clinical improvement.

## Conclusions

In conclusion, PEODDN is a rare skin disorder of unknown etiology, with characteristic clinical and histological findings. We reported an adult-onset case involving the right side of the trunk and upper extremity that responded well to treatment with acitretin. Accordingly, we recommend further evaluation of the effect of oral retinoids on PEODDN. Given the rarity of adult-onset presentations and the variable clinical morphology, clinicians should maintain a broad differential diagnosis when encountering linear dermatoses. Histopathological examination remains essential for accurate diagnosis, especially in atypical anatomical locations. Further case reports and studies are needed to better understand the natural history, associated conditions, and long-term response to systemic therapies.

## References

[1] Zade J, Jfri A, Nabatian A, Alajaji A, Geller L, Khorasani H (2025). Porokeratotic eccrine ostial and dermal duct nevus: a unique case treated with CO₂ laser. Clin Case Rep.

[2] Tejapira K, Suchonwanit P (2025). Porokeratotic eccrine ostial and dermal duct nevus: a report of rare late-onset solitary lesion. Clin Cosmet Investig Dermatol.

[3] Marsden RA, Fleming K, Dawber RP (1979). Comedo naevus of the palm--a sweat duct naevus?. Br J Dermatol.

[4] Abell E, Read SI (1980). Porokeratotic eccrine ostial and dermal duct naevus. Br J Dermatol.

[5] Bergman R, Lichtig C, Cohen A, Friedman-Birnbaum R (1992). Porokeratotic eccrine ostial and dermal duct nevus: an abnormally keratinizing epidermal invagination or a dilated, porokeratotically plugged acrosyringium and dermal duct. Am J Dermatopathol.

[6] Stoof TJ, Starink TM, Nieboer C (1989). Porokeratotic eccrine ostial and dermal duct nevus. Report of a case of adult onset. J Am Acad Dermatol.

[7] Masferrer E, Vicente MA, Bassas-Vila J, Rovira C, González-Enseñat MA (2010). Porokeratotic eccrine ostial and dermal duct naevus: report of 10 cases. J Eur Acad Dermatol Venereol.

[8] Wang NS, Meola T, Orlow SJ, Kamino H (2009). Porokeratotic eccrine ostial and dermal duct nevus: a report of 2 cases and review of the literature. Am J Dermatopathol.

[9] Kwon BR, Kim R, Byun JY, Choi YW, Choi HY, Lee MY (2021). A case of porokeratotic adnexal ostial nevus misdiagnosed as wart. Clin Case Rep.

[10] Yu HJ, Ko JY, Kwon HM (2004). Linear psoriasis with porokeratotic eccrine ostial and dermal duct nevus. J Am Acad Dermatol.

